# Arabidopsis *ASYMMETRIC LEAVES2* (*AS2*): roles in plant morphogenesis, cell division, and pathogenesis

**DOI:** 10.1007/s10265-021-01349-6

**Published:** 2021-10-19

**Authors:** Yasunori Machida, Takanori Suzuki, Michiko Sasabe, Hidekazu Iwakawa, Shoko Kojima, Chiyoko Machida

**Affiliations:** 1 Division of Biological Science, Graduate School of Science, Nagoya University, Nagoya, Aichi 464-8602 Japan; 2 Central Research Institute, Ishihara Sangyo Kaisha, Ltd., 2-3-1 Nishi-Shibukawa, Kusatsu, Shiga 525-0025 Japan; 3 Department of Biology, Faculty of Agriculture and Life Science, Hirosaki University, 3 Bunkyo-cho, Hirosaki, 036-8561 Japan; 4 Graduate School of Bioscience and Biotechnology, Chubu University, Kasugai, Aichi 487-8501 Japan

**Keywords:** Arabidopsis leaf development, AS2 bodies, Epigenetic regulation, Nucleolus, Ribosomal DNAs, Phylogenetic tree, AS2/LOB family

## Abstract

The *ASYMMETRIC LEAVES2* (*AS2*) gene in *Arabidopsis thaliana* is responsible for the development of flat, symmetric, and extended leaf laminae and their vein systems. AS2 protein is a member of the plant-specific AS2/LOB protein family, which includes 42 members comprising the conserved amino-terminal domain referred to as the AS2/LOB domain, and the variable carboxyl-terminal region. Among the members, *AS2* has been most intensively investigated on both genetic and molecular levels. AS2 forms a complex with the myb protein AS1, and is involved in epigenetic repression of the abaxial genes *ETTIN*/*AUXIN RESPONSE FACTOR3* (*ETT*/*ARF3*), *ARF4*, and class 1 *KNOX* homeobox genes. The repressed expression of these genes by *AS2* is markedly enhanced by the cooperative action of various modifier genes, some of which encode nucleolar proteins. Further downstream, progression of the cell division cycle in the developing organs is stimulated; meristematic states are suppressed in determinate leaf primordia; and the extension of leaf primordia is induced. AS2 binds the specific sequence in exon 1 of *ETT*/*ARF3* and maintains methylated CpGs in several exons of *ETT*/*ARF3*. AS2 forms bodies (designated as AS2 bodies) at nucleolar peripheries. AS2 bodies partially overlap chromocenters, including inactive 45S ribosomal DNA repeats, suggesting the presence of molecular and functional links among AS2, the 45S rDNAs, and the nucleolus to exert the repressive regulation of *ETT*/*ARF3*. The AS2/LOB domain is characterized by three subdomains, the zinc finger (ZF) motif, the internally conserved-glycine containing (ICG) region, and the leucine-zipper-like (LZL) region. Each of these subdomains is essential for the formation of AS2 bodies. ICG to LZL are required for nuclear localization, but ZF is not. LZL intrinsically has the potential to be exported to the cytoplasm. In addition to its nuclear function, it has been reported that AS2 plays a positive role in geminivirus infection: its protein BV1 stimulates the expression of *AS2* and recruits AS2 to the cytoplasm, which enhances virus infectivity by suppression of cytoplasmic post transcriptional gene silencing.

## Genetic roles of *AS2*, *AS1*, and modifier genes in leaf development

**Fig. 1:**
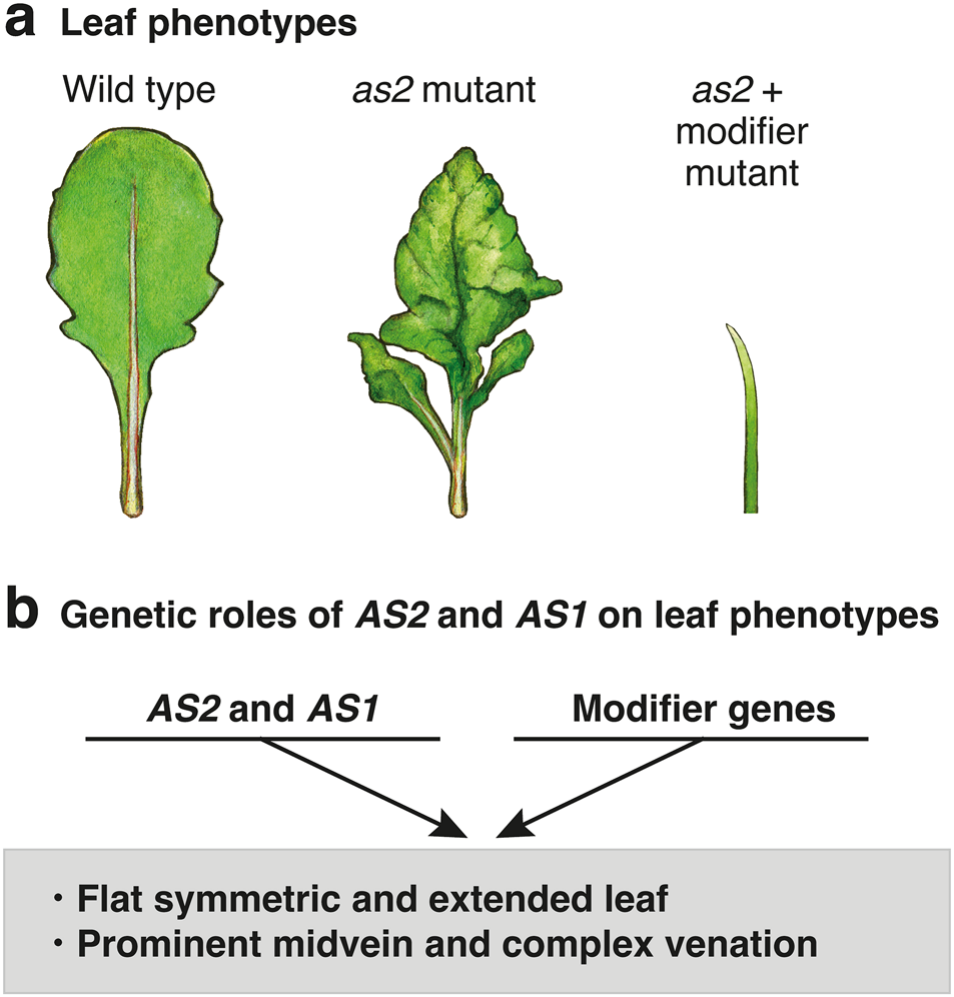
*AS2* and *AS1* are involved in the development of the flat and symmetrically extended leaf in Arabidopsis. **a** Leaf phenotypes of wild type (Col-0), the *as2-1* single mutant, *as2-1,* and modifier double mutant (illustrated by Moeko Machida). **b** Mode of actions of *AS2, AS1,* and modifier genes on leaf phenotypes

Molecular and genetic analyses of the *AS2* gene in *Arabidopsis thaliana* have shown that it is a key regulator for the development of flat symmetric leaves containing vascular bundles and fine networks of venation systems, the morphology of which seems to be evolved suitably for efficient photosynthesis (Fig. 1a) (Byrne et al. 2000; Ikezaki et al. 2010; Iwakawa et al. 2020; Machida et al. 2015; Ori et al. 2000; Semiarti et al. 2001; Serrano-Cartagena et al. 1999). The *as2* mutant generates shorter petioles, leaf lobes, and leaflet-like protrusions from the petioles of leaves in a bilaterally asymmetric manner (Fig. 1a) (Semiarti et al. 2001). The leaf lamina is often plump with a humped, wavy surface and reduced complexity of the leaf venation patterns (Semiarti et al. 2001). Similar abnormalities are observed in leaf lamina of the *as1* mutant leaves as well (Candela et al. 1999; Tsukaya and Uchimiya 1997), suggesting that *AS2* and *AS1* are involved in similar processes to develop flat symmetric and extended leaves and vascular systems consisting of the prominent midvein and complex fine networks of leaf venation. Many mutations that enhance phenotypes of *as2* and *as1* have been identified (Horiguchi et al. 2011; Ishibashi et al. 2012; Kojima et al. 2011; Luong et al. 2018; Matsumura et al. 2016; Pinon et al. 2008; Ueno et al. 2007; Yao et al. 2008), and see additional references summarized by Machida et al. (2015). We designated the causative genes as “modifiers” for *as2* and *as1* mutants. Double mutants containing both *as2* (or *as1*) and each of the modifier mutations commonly generate pointed and/or filamentous (rod-shaped) leaves surrounded by abaxialized epidermal cells, in which the development of phloem and xylem cells is not obvious (Ishibashi et al. 2012; Matsumura et al. 2016) (Fig. 1a, b). Genes involved in a wide variety of biological processes (e.g., biogenesis of small RNAs, chromatin modification and remodeling, biogenesis of ribosomes, DNA replication and repair, and formation of proteasomes) have been identified as modifiers (Iwakawa et al. 2020; Machida et al. 2015). Since the details of these mutants have been previously described in the above reviews, we will herein introduce two common features of these mutants. Modifier mutations are characterized as being either weak mutations of an essential gene or mutations in some genes in a family including multiple members. Another interesting feature is that many of the modifier proteins are localized in the nucleus, the nucleolus, and/or accumulated during mitosis of the cell division cycle. These findings imply that AS2 (or AS1) and the modifier proteins might interact cooperatively in the nucleus and/or the nucleolus to be involved in the development of flat symmetric leaves (Fig. 1b), as well as in complex vein systems in leaves. Conventional transcription factors, however, have been excluded from groups of modifiers.

Chemicals that enhance the phenotypes of *as2* and *as1* single mutants have been also identified. They include two specific inhibiters against histone deacetylases, trichostatin A (TSA) and 4-(dimethylamino)-N-[6-(hydroxyamino)-6-oxohexyl]-benzamide (CAY) (Ueno et al. 2007); berberine (a benzylisoquinoline alkaloid), which acts to bind guanine-rich triplex or quadruplex DNA and possesses various biological activities, as well as has been used in medicines (Nakagawa et al. 2012); and hydroxyurea, a known DNA replication inhibitor (Luong et al. 2018). In addition, by screening for natural compounds that stimulate the formation of filamentous leaves in the *as2* background, we have found more compounds that act as modifier mutations (our unpublished observations).

## Repressive controls of abaxial-determining *ARFs *and class 1 *KNOXs *by *AS2* and *AS1* are critical for the development of flat symmetric and extended leaves

The *AS2* gene was identified and its genetic, molecular, and cellular biological roles in leaf development have been intensively investigated for over 20 years (Iwakawa et al. 2002, 2007, 2020; Machida et al. 2015; Semiarti et al. 2001). Transcription of *AS2* starts at the globular stage of embryogenesis, and its transcripts are accumulated in protoderm cells on the adaxial side of cotyledonary primordia at the heart stage (Iwakawa et al. 2002).

**Fig. 2:**
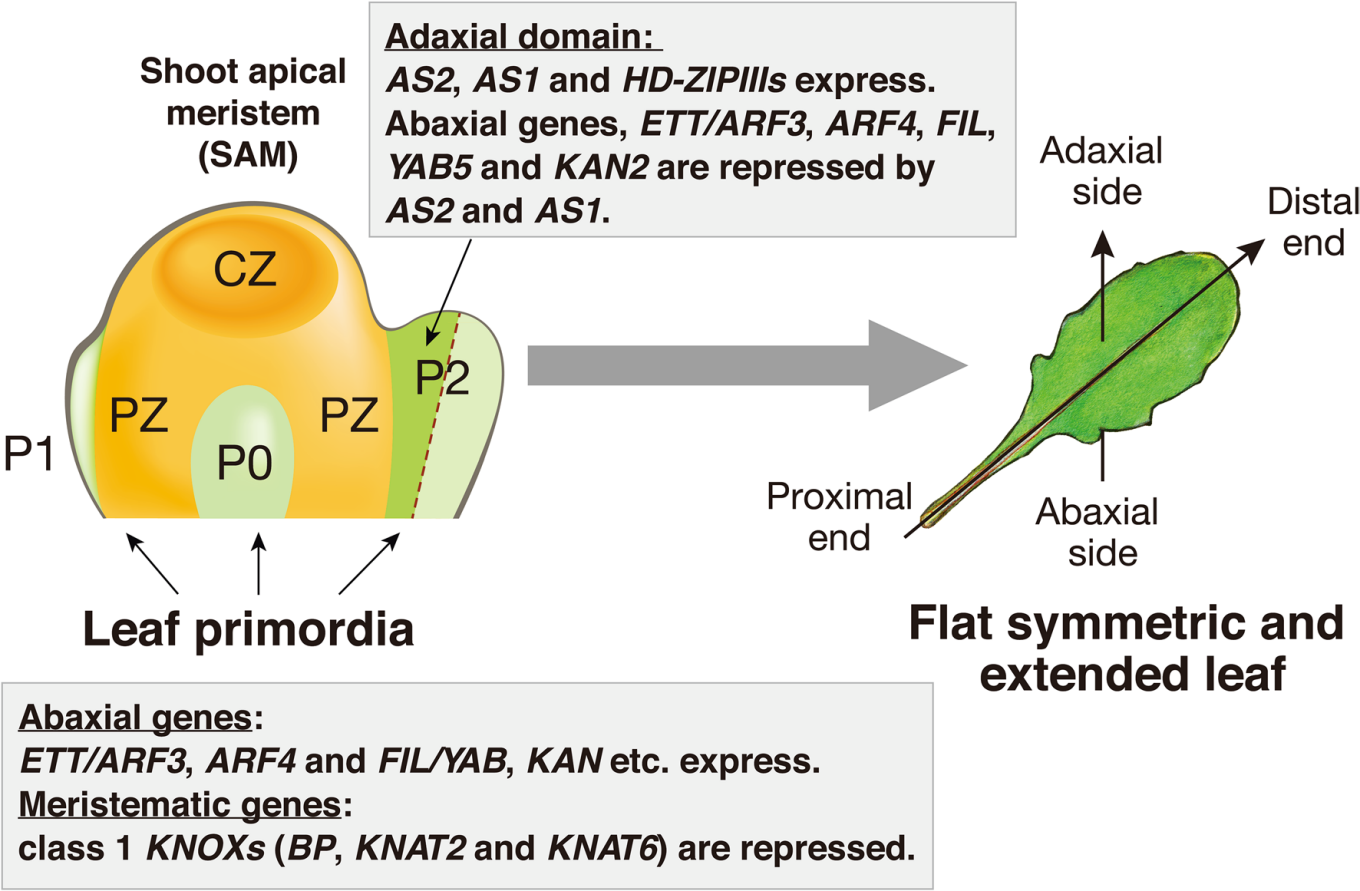
Alteration of expression of genes that are involved in abaxial-adaxial patterning during progression of the developmental stages of the leaf in Arabidopsis. P0, P1, and P2 represent the developmental stages of leaf primordia from the earliest P0. In the peripheral zone (PZ) of the shoot apical meristem (SAM) that is characterized by the expression of class 1 *KNOX* genes (*BP, KNAT2, KNAT6*), a leaf primordium is born by repressing these *KNOX* genes. At the early stages (P0-P1), the abaxial-determining genes, such as *ARFs, FIL/YAB,* and *KAN* are first expressed. Subsequently, *AS2* and *AS1* are expressed in the presumptive adaxial domain in the leaf primordium (stage P2), and repress the expression of the abaxial-determining genes, which leads to expression of the adaxial-determining genes, *HD-ZIPIIIs,* differentiation of the adaxial domain, and establishment of the abaxial-adaxial pattern. The repression of class 1 *KNOX* genes listed here also plays a role in the extension of leaves

As shown in Fig. 2, after germination, the *AS2* transcript is only faintly detected in the shoot meristem, and the pattern of its distribution around leaf primordia (P0-P1 stages) is obscure. During development of the primordia of cotyledons and leaves, *AS2* expression is detected in epidermal layers of their adaxial domains (P2 stage) (Iwakawa et al. 2007). *AS1* transcripts are detected in wide areas in the leaf primordia at very early stages, and distribution of the transcripts focus onto central regions of the leaf primordia, including vascular bundles, as the leaf primordia grow.

The process of leaf differentiation is initiated by expression of the abaxial-domain determining genes, *KAN*s, *FIL*/*YABs,* and *ETT*/*ARF3*, in the earliest stages of the leaf primordia (P0 and P1) (Fig. 2). Subsequently, a group of genes including *AS2, AS1*, and *HD-ZIPIII*s are expressed in the presumptive adaxial area in the leaf primordium at the P2 stage to develop the adaxial domain. The expression of *AS2* and *AS1* is consistently detected during development of young cotyledons and leaves (Iwakawa et al. 2007) to support flat symmetric and extended organs.

**Fig. 3:**
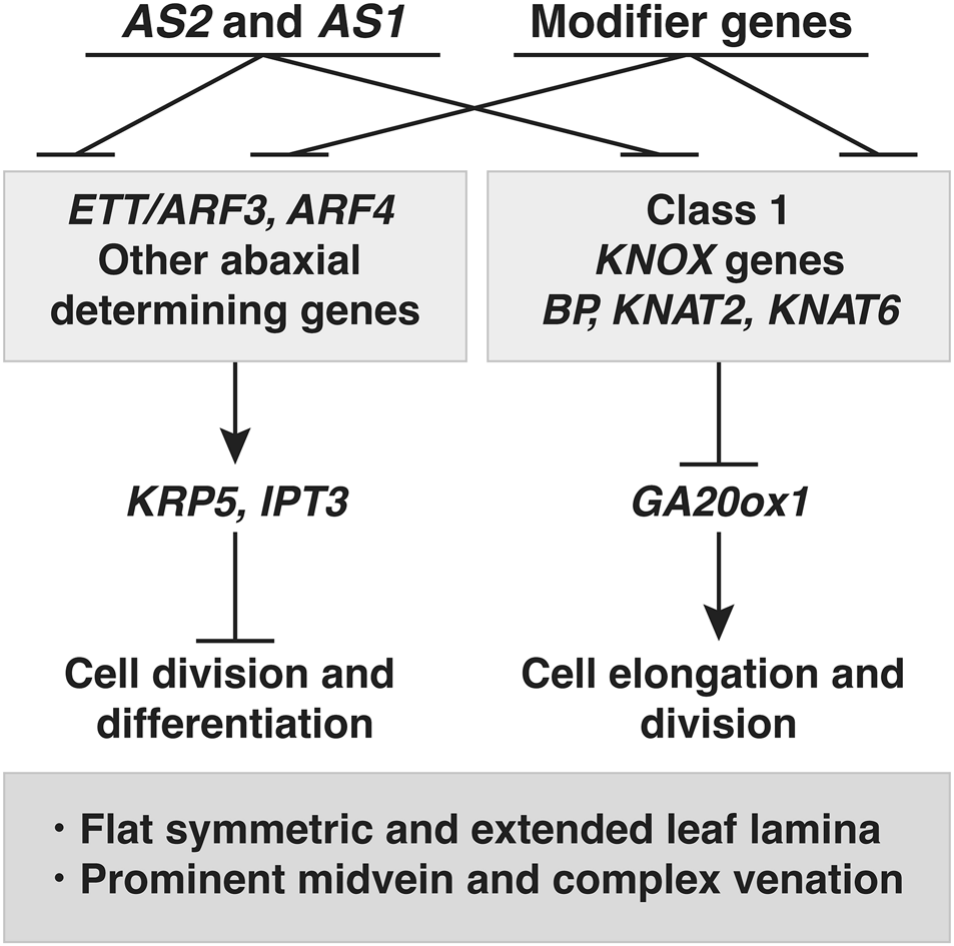
Gene network by *AS2, AS1* and modifier genes for leaf morphology. AS2 and AS1 directly repress the downstream genes shown in boxes. *ETT/ARF3* and class 1 *KNOX* genes regulate transcription of the further downstream genes, *KRP5; IPT3;* and *AtGA20ox1,* respectively

As conceptually illustrated in Fig. 3, AS2 and AS1 directly repress the transcription of *ETT/ARF3* by binding to its promoter region (Iwasaki et al. 2013), and they also indirectly repress the expression of both *ETT*/*ARF3* and its closely related *ARF4* genes by degradation of their *ARF* transcripts by the RNA silencing mechanism including biogenesis of miR390 and tasiR-ARF (Iwasaki et al. 2013; Takahashi et al. 2013). Phenotypic abnormalities of *as2* leaves are restored by the introduction of *ett* and *arf4* mutations, which provide the genetic evidence that *AS2* represses the expression of *ETT*/*ARF3* (Iwasaki et al. 2013). Genetic analyses with *as2* and *as1* mutants imply that suppression of the abaxial genes by AS2 and AS1 in the presumptive adaxial area is critical to ensure differentiation of the adaxial domain in the subsequent step. It might be inevitable for the plant to form balanced adaxial and abaxial domains in order to create a leaf as a flat and symmetrically extended organ. In addition, as described in the first section, it should be worth pointing out that such transcriptional repression of *ETT*/*ARF3* and *ARF4* is further achieved by cooperative action with various modifier genes, such as *NUC1, RH10,* and *RID2*, the proteins of which are localized to the nucleolus (Matsumura et al. 2016).

AS2 and AS1 also repress the transcription of three class 1 *KNOX* genes, *BREVIPEDICELLUS (BP)*, *KNAT2,* and *KNAT6* (Fig. 3) (Byrne et al. 2000; Ikezaki et al. 2010; Semiarti et al. 2001) by binding to their respective promoter regions (Guo et al. 2008). This repression results in leaf elongation in the proximal–distal direction (Fig. 2) (Machida et al. 2015; Semiarti et al. 2001).

The AS2 protein is an untypical conventional transcription factor, as described below, and a member of the AS2/LOB protein family that is only present in the plant kingdom. *AS1* encodes the myb domain protein. AS2 and AS1 proteins form a complex (AS2-AS1) (Guo et al. 2008; Yang et al. 2008), that represses the transcription of the downstream genes introduced in the next section. AS1 is involved in histone H3 modification, which induces chromatin condensation of the *BP* gene region to repress the expression of this gene (Li et al. 2016). In addition to this repression system, as described in the following section, we have proposed that AS2-AS1 might be involved in a novel type of epigenetic repressive control of the downstream gene *ETT*/*ARF3* (Iwasaki et al. 2013; Vial-Pradel et al. 2018).

## Genes downstream of *ARFs *and class 1 *KNOX*s

*ETT*/*ARF3* and *ARF4* positively control both the *KIP-RELATED PROTEIN 5* (*KRP5*) gene and the *ISOPENTENYL-TRANSFERASE 3* (*IPT3*) gene (Fig. 3) (Takahashi et al. 2013). Within this entire pathway from the AS2-AS1, the expression of *KRP5* and *IPT3* could be suppressively controlled. Since *KRP5* encodes a CDK inhibitor, a negative regulator of the cell division cycle, AS2-AS1 seems to stimulate the progression of M phase during leaf formation. The repressive control of *IPT3,* which mediates the biosynthesis of cytokinin, seems to play a critical role in the stabilization of the determinate states of differentiated leaf cells to form flat symmetric leaves.

Repressive control of expression of the gibberellin (GA) biosynthesis gene encoding GA 20-oxidase by the class 1 *KNOX* gene was first reported with the tobacco plant (Sakamoto et al. 2001). In *A. thaliana*, AS2-AS1 represses the transcription of three *KNOX* genes (*BP*, *KNAT2,* and *KNAT6*), but not *STM* (Semiarti et al. 2001). These three *KNOXs* redundantly function in repressing transcription of the *AtGA20ox1* gene, which is involved in cell elongation and division in leaves (Figs. 2, 3) (Ikezaki et al. 2010).

## AS2 is a member of the AS2/LOB protein family and *AS2* acts as a single-copy gene

**Fig. 4:**
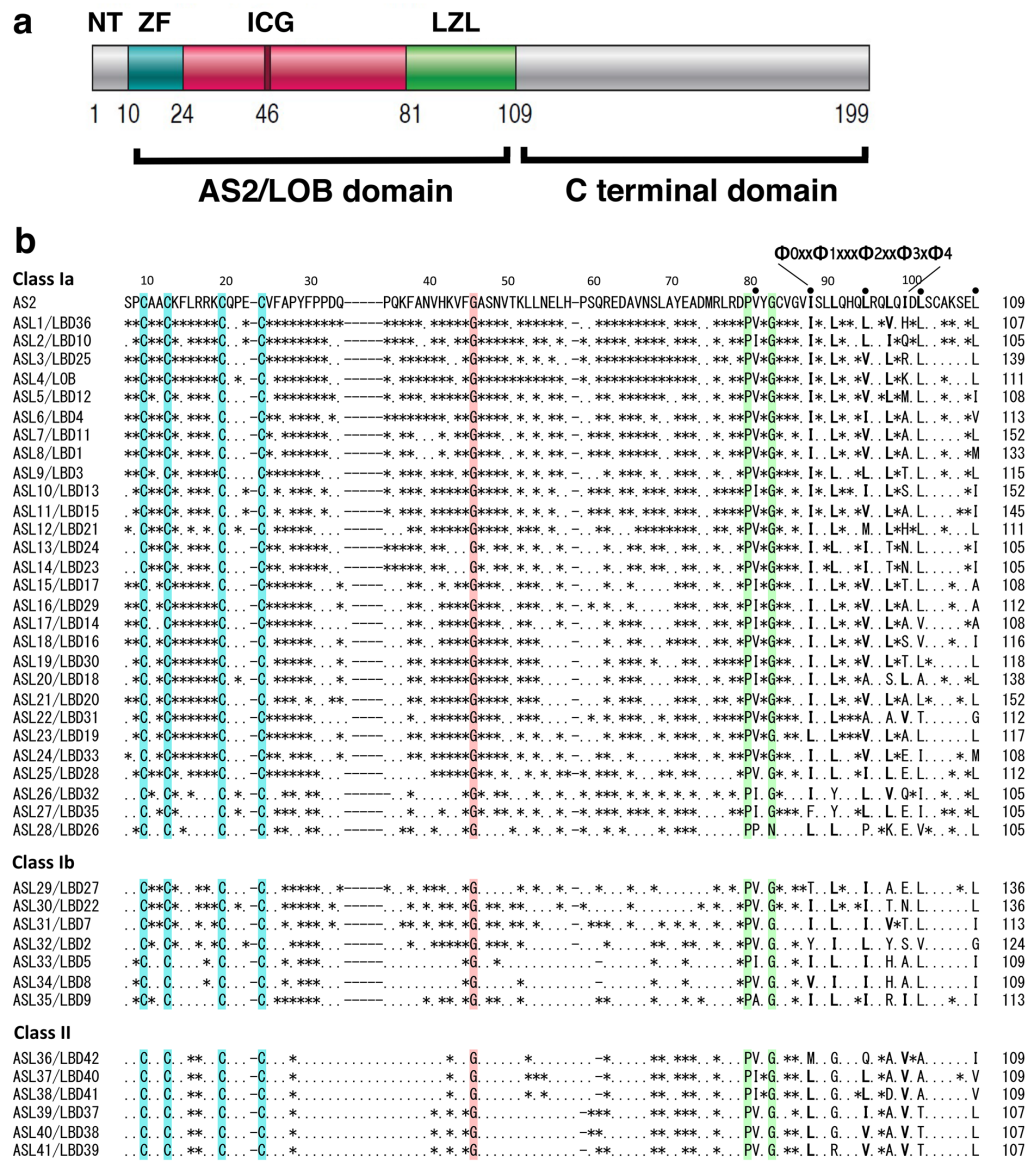
Characteristic structures of AS2 and AS2/LOB family proteins. **a** Organization of domains and subdomains in the AS2 protein. NT, ZF, ICG, and LZL represent the N-terminal region, the zinc-finger motif, the internally conserved-glycine region, and the leucine-zipper-like region, respectively. **b** Comparison of the predicted amino acid sequence of the AS2 domains of AS2 and those of AS2-like proteins (ASLs). Residues that are conserved in all and 41/42 members of the family are colored. Asterisks, dots, and dashes in sequences of family members represent residues identical to those of AS2, different from those of AS2, and gaps, respectively. The AS2 sequence from residue 8 to residue 109 is aligned with those from corresponding sequences of the members (ASLs; see text). According to this nomenclature, members with close numbers have high amino acid sequence identity with each other. Dots and Φ0xxΦ1xxxΦ2xxΦ3xΦ4 above AS2 indicate the position of the hydrophobic residues conserved in LZL. See Sects. 6 and 8 in text

AS2 is a member of the AS2/LOB protein family that consists of 42 predicted proteins in *A. thaliana* (Fig. 4) (Iwakawa et al. 2002; Shuai et al. 2002), and is the first family member to have its genetic properties associated with its molecular characteristics (Iwakawa et al. 2002; Semiarti et al. 2001). AS2 protein has the AS2/LOB domain in the amino-terminal half (Fig. 4a), the sequence of which is more or less conserved among the family members (Fig. 4b) (Iwakawa et al. 2002; Matsumura et al. 2009). These members, other than AS2, are numbered in order from the one in which the amino acid sequence of the AS2/LOB domain is closest to the sequence of that in the AS2 protein [e.g., the closest member is named AS2-like protein 1 (ASL1 for short)] (Iwakawa et al. 2002). These members are also called Lateral Organ Boundary (LOB) domain (LBD) proteins (Shuai et al. 2002); however, to date, the genetic function of LOB (ASL4) remains unknown. Forty-two members are divided into two classes based on the similarity of the amino acid sequences of the AS2/LOB domains (Fig. 4b), Class I (AS2 and ASL1-ASL35) and Class II (ASL36-ASL41): Class I is further divided into two sub-classes Class Ia (ASL1-ASL28), and Class Ib (ASL29-ASL35).

**Fig. 5:**
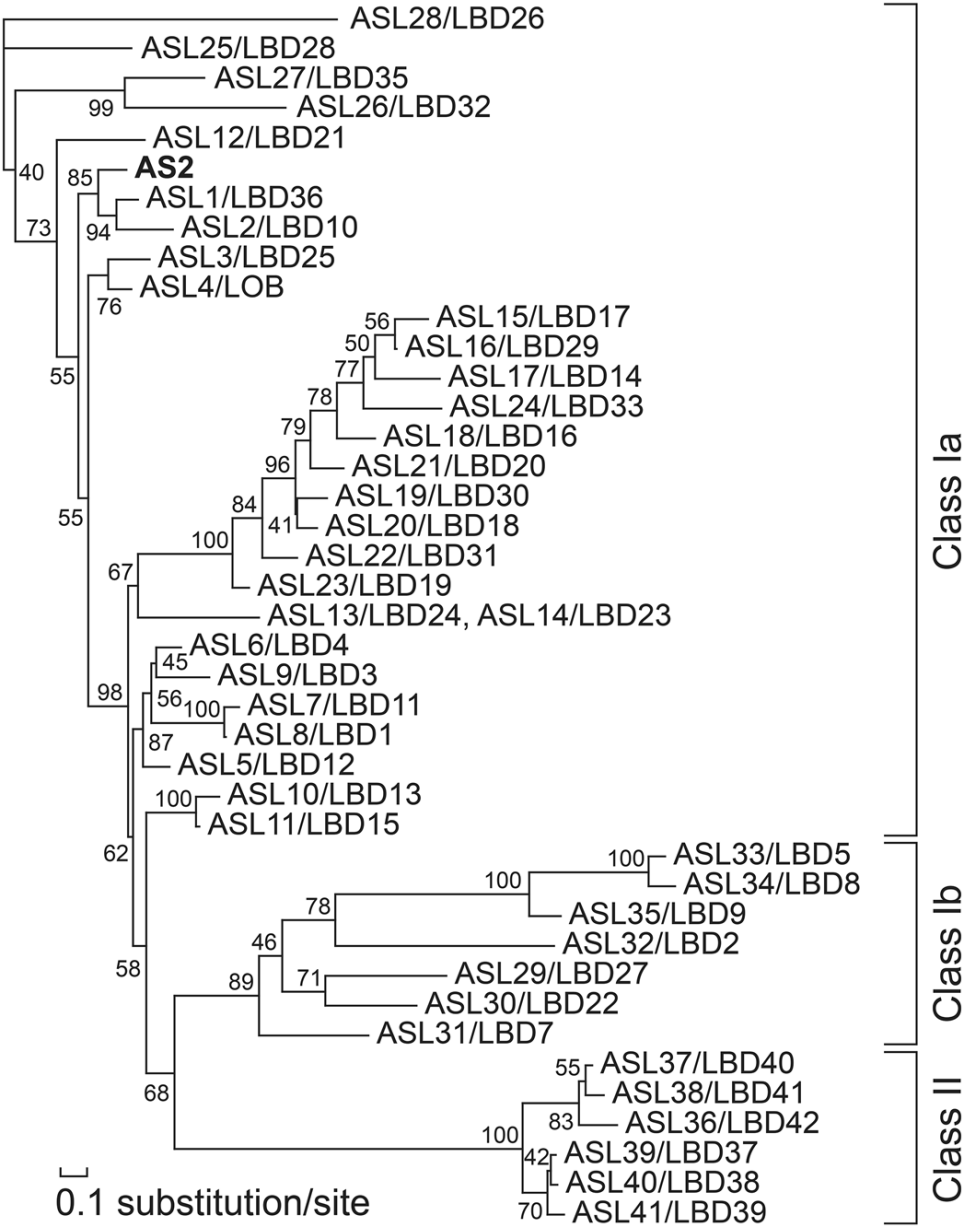
An unrooted maximum-likelihood tree for 42 members of the AS2/LOB family of proteins from Arabidopsis, as generated by a local rearrangement search. Numbers on branches represent local bootstrap values, which were calculated with the ProtML program. The length of each horizontal branch is proportional to the estimated evolutionary distance. The brackets on the right indicate the classification of members of the AS2/LOB family as shown in Fig. 4b. The NJdist and ProML programs in MOLPHY package version 2.3b3 were used (https://www.ism.ac.jp/ismlib/eng/ismlib/softother.html#molphy). The panel was reproduced and modified from a previous report (Iwakawa et al. 2002)

Figure 5 shows the phylogenetic tree of the AS2/LOB protein family of *A. thaliana*, a modification of the tree of the AS2 family previously reported (Iwakawa et al. 2002). Two types of nomenclature (ASLs and LBDs) are listed side by side and such nomenclature with ASLs should be advantageous for discussing relationships between the phylogeny and developmental functions of members in the AS2/LOB protein family. For example, at least four known proteins involved in auxin-induced lateral root formation are named ASL18/LBD16, ASL16/LBD29, ASL20/LBD18, and ASL24/LBD33; ASL15/LBD17 is also proposed to play a similar role (Berckmans et al. 2011; Goh et al. 2012; Lee et al. 2009; Okushima et al. 2007). All of them are located in the close clades of sub-class Class Ia in the phylogenetic tree. During pollen development, ASL1/LBD36, ASL2/LBD10, and ASL3/LBD25, all of which belong to the close narrow clades of the tree, control asymmetric cell division during pollen development (Kim et al. 2015, 2016). Interestingly, another set of ASL members (SCP/ASL29/LBD27 and ASL30/LBD22 in a small clade) are also involved in the same single process, but at different steps during the progression of pollen development (Kim et al. 2015, 2016; Oh et al. 2010). Note that ASL1/LBD36, ASL2/LBD10, and ASL3/LBD25 belong to Class Ia, and that SCP/ASL29/LBD27 and ASL30/LBD22 belong to Class Ib. Use of the ASL nomenclature might provide an edge to discussion of evolutionary developmental biology (evo-devo) of the AS2/LOB family.

The AS2/LOB domain consists of three subdomains [the zinc-finger (ZF) motif, the internally conserved-glycine (ICG) region, and the leucine-zipper-like (LZL) region]. The ZF motifs in all members have four perfectly conserved cysteine residues (at positions 10, 13, 20, and 24, in AS2), although limited sequence divergences are found among these cysteine residues. The ZF motif of AS2 is essential for binding to the specific DNA sequence in the downstream target *ETT*/*ARF3* genome as described in the next section (Vial-Pradel et al. 2018). In other subdomains, three amino acid residues are completely, or nearly completely, conserved in all of the family members. In the ICG regions, the glycine residues (at position 46 in AS2) are conserved among all members. The LZL regions are considered to be involved in protein–protein interactions to form homo and/or hetero multimers. The proline residues (at position 80 in AS2) at the border between the ICG and LZL regions are conserved in all members. Interestingly, the glycine residue (at position 83 in AS2) is conserved in 41 members, other than ASL28/LBD26, out of 42. These perfectly conserved residues might play common roles in molecular functions as proteins of the AS2/LOB family. There are also amino-terminal regions (designated NTs) that are variable in length among these members, although little is known about their roles (Luo et al. 2012; Matsumura et al. 2009).

**Fig. 6:**
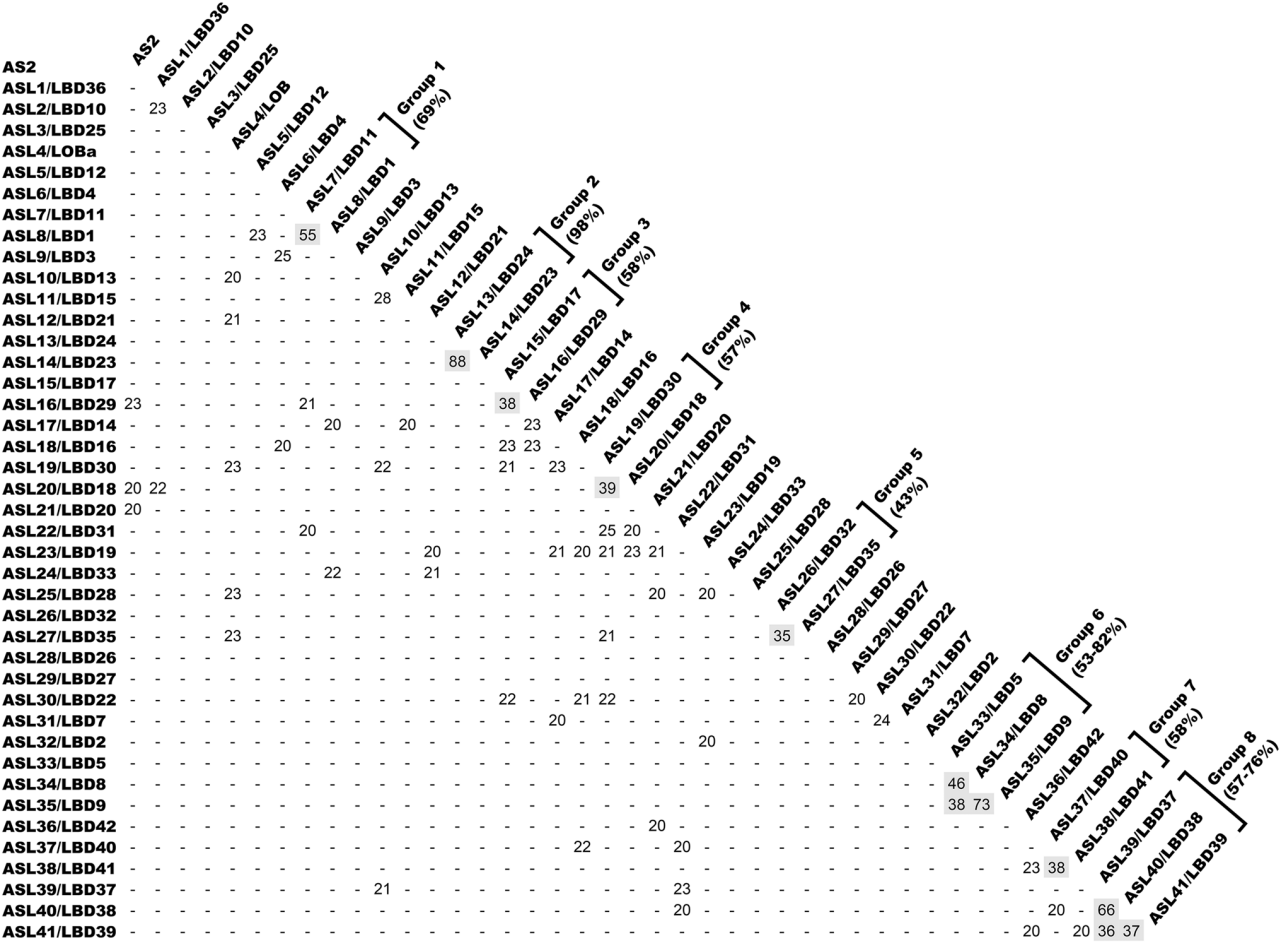
Comparison of the amino acid sequences of the C-terminal halves of predicted proteins of the AS2/LOB family. Each number indicates the identity, as a percentage, between the predicted amino acid sequences of indicated proteins. Percentages greater than 20% are indicated and those less than 20% are marked with −. Percentages greater than 35% are shaded. Numbers in percentages under group designations indicate the identities between entire amino acid sequences, showing that two members in each group have high similarity in entire amino acid sequences. These analyses show that close numbers in ASLs indicate sequence similarities

A comparison of deduced amino acid sequences of carboxy-terminal halves in the family members shows that no member exhibits a significant similarity to that of AS2 (Fig. 6) (Matsumura et al. 2009). Since the *as2-4* allele had a frame shift mutation in this region (Iwakawa et al. 2002), and the phenotype due to this allele is similar to those due to other *as2* alleles (Semiarti et al. 2001), it seems that the C-terminal half is essential for the functions of AS2. The domain swapping experiment between AS2 and each of ASL1, ASL2, and ASL3, the closest members of AS2, has shown that the AS2/LOB domain of AS2 cannot be replaced by those of these closest members, revealing that a few dissimilarities among amino acid residues in the AS2/LOB domain of AS2 and those of these members are critical for the functions of AS2 in leaf development (Matsumura et al. 2009). These sequence analyses and experimental results suggest that *AS2* acts as a single-copy gene from the viewpoint of leaf morphogenesis in *A. thaliana*.

## AS2 protein binds exon 1 of *ARF3* and maintains methylated CpGs in other exons

The AS2 protein binds to synthetic double-stranded DNAs containing the sequence GCGGCG as the core motif (Husbands et al. 2007). AS2 forms an immuno-complex with *ETT*/*ARF3* genomic DNA covering its exon 1 (Iwasaki et al. 2013), which includes the CGCCGC/GCGGCG sequence. Our in vitro binding experiments have shown that the AS2 protein binds to the DNA segment in exon 1 of the *ETT*/*ARF3* gene, and that the CGCCGC sequence is critical, but not sufficient, for that binding (Vial-Pradel et al. 2018). The AS2/LOB domain is sufficient for the binding to the DNA segment, and its ZF motif plays an essential role in that binding.

Genome-wide methylome analysis shows that CpG dinucleotides in exons 6, 9, and 10 in the *ETT*/*ARF3* locus are methylated in the Col-0 plant (Cokus et al. 2008; Zhang et al. 2006). Exon 6 contains six CpGs, which are highly methylated depending on METHYLTRANSFERASE1, an Arabidopsis ortholog of the vertebrate DNMT1, and AS2 and AS1 are involved in the maintenance of these methylated CpGs (Iwasaki et al. 2013). The presence of the ZF motif in AS2 would be reminiscent of the CxxC-type zinc finger in DNMT1. Modifier genes such as *NUC1* and *RH10,* mentioned in Sect. 2 (Figs. 1, 3), are also involved in the maintenance of the methylation state of CpG in exon 6 (Vial-Pradel et al. 2018). These modifier genes encode nucleolar proteins (Kojima et al. 2007; Matsumura et al. 2016; Petricka and Nelson, 2007; Pontvianne et al. 2007). These results imply that the maintenance of CpG methylation by AS2-AS1 might be related to the cooperative repression of *ETT*/*ARF3* expression with these nucleolar modifier proteins.

Roles of the molecular events mediated by AS2-AS1 and nucleolar proteins in the repressive control of the *ETT*/*ARF3* gene need to be experimentally investigated.

## AS2 forms perinucleolar bodies in interphase cells that partially overlap with chromocenters containing rDNA repeats

**Fig. 7:**
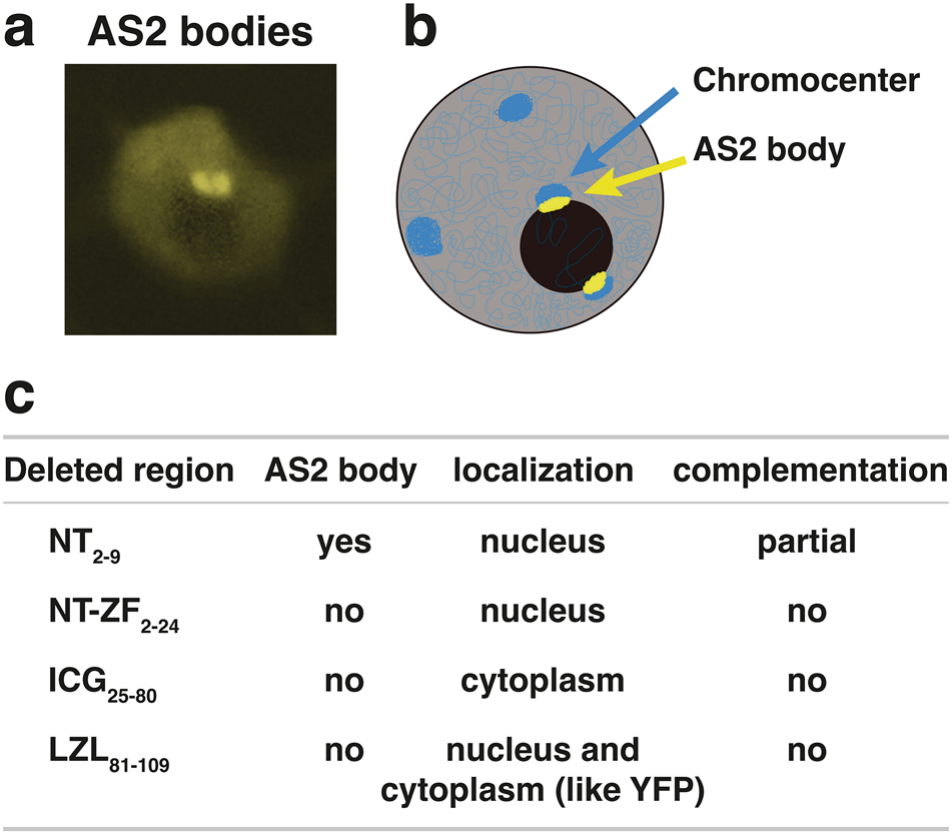
AS2 bodies at the periphery of the nucleolus in the interphase cell of the leaf primordium, where chromocenters containing 45S rDNA repeats are localized. **a** Two AS2 bodies are visible by fluorescence due to YFP (yellow fluorescent protein) at the periphery of the nucleolus in cells of the leaf primordium of the AS2-YFP-transformed Arabidopsis. The dark area corresponds to the nucleolus (reproduced from Ueno et al. 2007). **b** Schematic image of the AS2 bodies (yellow) and chromocenters (blue). It has been reported that interphase nuclei from cells of Arabidopsis contain up to 10 chromocenters, which locate near the nuclear periphery and the nucleolus (Fransz et al. 2002). Only several chromocenters were, however, usually visible in an optical section under single focuses by our observations. See the top panel in Fig. 8. **c** Indicated subdomains (see Fig. 4a) were deleted in these mutant proteins. Subscripts represent positions of deleted amino acid residues in the mutants. Mutant proteins were fused to YFP. These fused constructs were tested for abilities to form AS2 bodies. Patterns of subcellular localization and abilities of fused constructs to complement *as2-1* phenotypes were also examined. Panels **a** and **c** were reproduced and modified from references by Ueno et al. (2007) and Luo et al. (2020), respectively

**Fig. 8:**
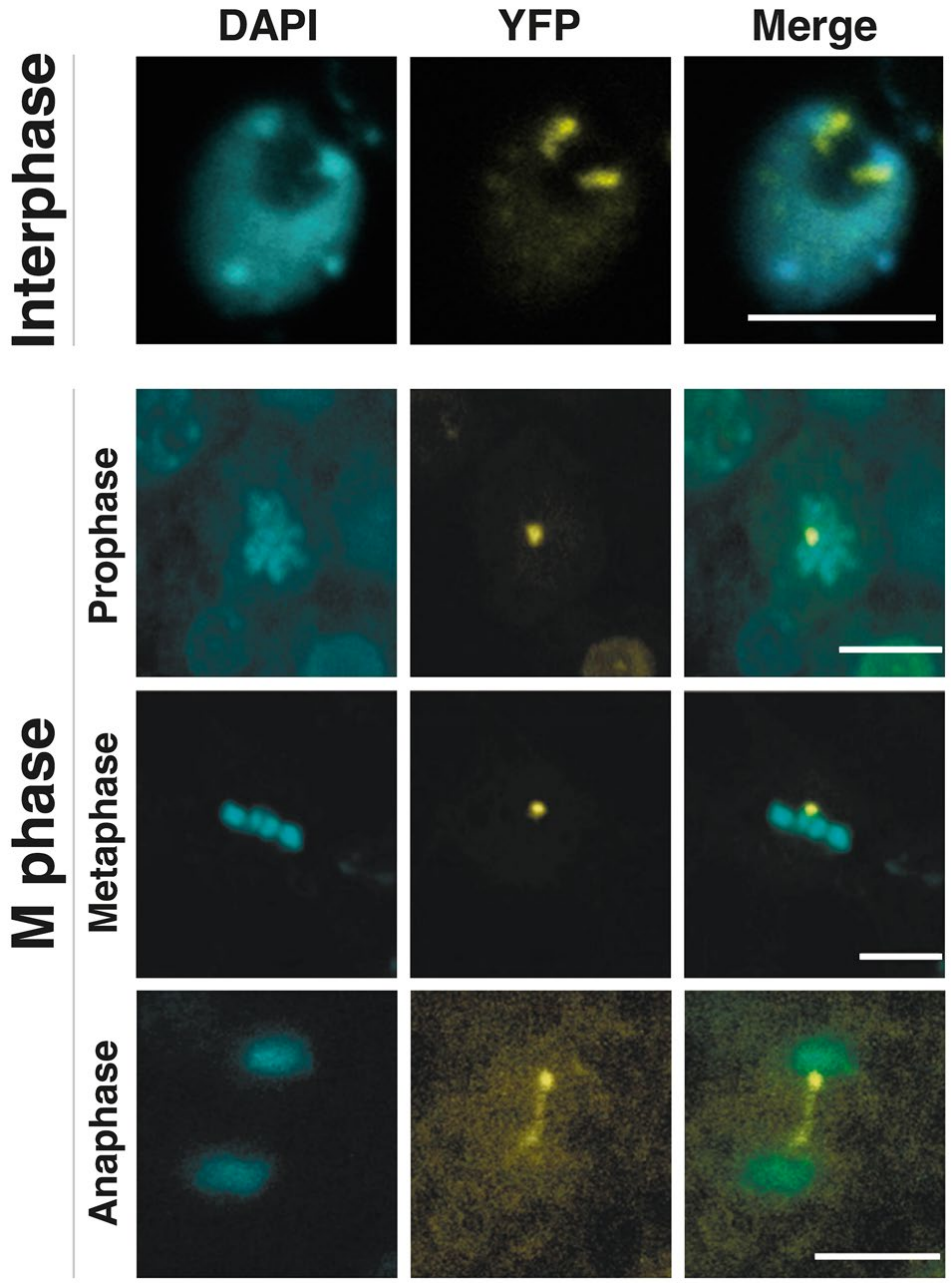
AS2 bodies exhibit dynamic movement during the M phase progression of cells in leaf primordia. Patterns of subcellular localization of AS2-YFP proteins (yellow) were observed in epidermal cells of the adaxial domain in leaf primordia of the transgenic Arabidopsis plants. Nuclei and chromosomes were stained with 4,6-diamidino-2-phenylindole (DAPI) (cyan). Under a single focus, one AS2 body is often observed in most single cells undergoing prophase to metaphase of M phase, although the average number of the bodies in a cell during interphase is approximately 1.9 (see Text). Anaphase bridge-like structures are visualized during the progression from anaphase to telophase. Panels were reproduced and modified from a previous report (Luo et al. 2020). Bars = 5 µm

By using the estradiol-inducible expression system for AS2-fused yellow fluorescence protein (AS2-YFP), we investigated the subcellular localization of the AS2 protein in cells of a transgenic Arabidopsis plant and cultured cell lines of Arabidopsis and tobacco. We reported that AS2 forms granules (designated as AS2 bodies) at the periphery of the nucleolus in interphase cells in the epidermis of the adaxial domains of cotyledonary and leaf primordia (Figs. 7a, 8), although some amounts of AS2-YFP are present in the nucleoplasm of these cells (Luo et al. 2012; Ueno et al. 2007). As illustrated in Fig. 7b, AS2 bodies are variable in shape and size; appear to be rough spheres or beans; and themselves also appear as aggregates of smaller particles. They are, however, not completely random in wild type *A. thaliana* (Col-0), although fine morphological and quantitative analyses have yet to be performed. The average number of AS2 bodies is calculated to be approximately 1.9 per YFP-positive interphase cell in leaf primordia of the wild-type plant of *A. thaliana* Col-0 (Luo et al. 2020).

The experiment with the glucocorticoids receptor (GR)-fused AS2 and dexamethasone shows that the migration of AS2 into the nucleus is essential for its role in leaf development (Ueno et al. 2007). It is, however, difficult to identify clearly the nuclear localization signal (NLS) in the AS2 sequence. Although the RRK sequence in the ZF motif (Fig. 4a, b) is proposed to function in nuclear localization (Chen et al. 2019), our result (Fig. 7c) shows that the deletion construct NT-ZF2-24 is not a formed AS2 body, but is localized to the nucleus, showing that the RRK cluster is not involved in the nuclear localization of AS2, and that the ICG and the LZL regions encompassing 85 amino acid residues are sufficient for the nuclear localization. An obvious NLS sequence is, however, absent from these regions, suggesting the involvement of a certain unidentified mechanism in the nuclear localization of AS2.

As shown in Figs. 7b and 8, AS2 bodies overlap partially with the chromocenters, distinct chromatin structures that consist of condensed heterochromatins containing repeats of the ribosomal DNA (45S rDNA encoding 18S, 5.8S, and 28S rRNAs) (Luo et al. 2020). In the diploid genome of *A. thaliana* (Col-0), there are approximately 1500 copies of the 45S rDNA gene on chromosomes 2 and 4, which exist as tandemly repeated sequences on each of the chromosomes (Copenhaver and Pikaard, 1996) . Two chromocenters corresponding to chromosome 4 in a diploid cell are detected at the periphery of the nucleolus, in which the 45S rDNA genes should be transcriptionally inactive (Pontvianne et al. 2010). The observation that AS2 bodies are overlapped with two nucleolar chromocenters in *A. thaliana* (Col-0) (Luo et al. 2020) predicts that a role of AS2 bodies might be related to the repressive regulation of *ETT*/*ARF3,* such as the inactivation of the 45S rDNA genes in the chromocenters. As mentioned in Sect. 5, AS2 binds to exon 1 of the *ETT*/*ARF3* gene and is involved in the maintenance of gene body methylation in its exon 6. These molecular events might also be involved in the repressive regulation of the gene expression at the nucleolar periphery.

The AS2/LOB domain, which is essential for binding to *ETT*/*ARF3*, is necessary and sufficient for the formation of AS2 bodies. We tested deletion mutants of the NT2-9, NT-ZF2-24, ICG25-80, and LZL81-109 regions (Fig. 4a) for their abilities to form AS2 bodies, to control subcellular localization, and to complement the mutant phenotype of *as2-1* (Fig. 7c). Results of those experiments showed that deletion constructs without one of the NT-ZF2-24, ICG25-80, and LZL81-109 regions are inactive to form AS2 bodies. Therefore, all of the ZF10-24 motif, ICG25-80, and LZL81-109 regions are essential for the formation of AS2 bodies. The finding that the NT-ZF2-24 deletion is localized to the nucleoplasm suggests that the ZF motif, which is essential for the binding to exon 1 of *ETT*/*ARF3*, is not responsible for the nuclear localization of AS2, but that it participates in the perinucleolar localization of AS2 and/or in the formation of AS2 bodies. It is likely that the LZL region is involved in interactions with AS2’s own proteins and/or other associating molecules. In addition, none of the mutants that did not form AS2 bodies could recover the *as2-1* phenotype. These results indicate that the formation of AS2 bodies is tightly correlated with the functioning of AS2 in normal leaf development (Luo et al. 2020).

The observation that the deletion construct ICG25-80 is exclusively localized to cytoplasm (Fig. 7c) was unexpected. The deletion construct LZL81-109 is detected in both the nucleoplasm and the cytoplasm, a localization pattern similar to that of the YFP protein, itself. These observations imply that signal(s) controlling nuclear localizations of AS2 might be present in the region covering both ICG and LZL. Interestingly, the LZL region includes the ISLLQHQLRQLQI sequence in residues from 88 to 100 (Fig. 4b), which closely resembles the Φ0xxΦ1xxxΦ2xxΦ3xΦ4 (Φ: hydrophobic residues) sequence proposed as the traditional consensus pattern for the nuclear export signal (NES) (Fung et al. 2015; Luo et al. 2020). The LZL region might function in exporting AS2 from the nucleus to the cytoplasm. The ICG region (residues 25–80) might play an inhibitory role by interacting with LZL in exporting AS2 to the cytoplasm. Similar Φ-rich sequences corresponding to the region from Φ0 to Φ3 are present in 23 (ASLs1-11, ASLs15-19, and ASLs21-27) out of 28 members of the class 1a subclass (Fig. 4b) (Iwakawa et al. 2002; Matsumura et al. 2009). Sequences similar to the Φ0xxΦ1xxxΦ2 consensus pattern are also found in the LZL region of Class Ib; however, sequences similar to these are not present in class II.

It would also be interesting to point out that the mutation of *NUCLEOLIN1* (*nuc1*) affects the number and distribution patterns of AS2 bodies in the nucleolus (Luo et al. 2020). The number of AS2 bodies is elevated: smaller sized bodies are found; and some bodies did not co-localize with the perinucleolar chromocenters.

Since NUC1 is one of the abundant proteins in the nucleolus and is involved in various molecular events, such as the processing of rRNA precursors, both the formation and the maintenance of AS2 bodies is presumed to be under the control of the global molecular architecture and interactions within the nucleus.

## AS2 bodies are consistently observed in mitotic cells

In addition to interphase cells (uppermost panel of Fig. 8), we examined AS2 bodies during the M phase of the cell cycle in leaf primordial cells of *A. thaliana* plants, its cultured cell line MM2d (Luo et al. 2020), and tobacco cell line BY2 (Luo et al. 2012). One of the characteristic features of AS2 bodies in relationship to the cell cycle is that they are also observed after the disappearance of the nuclear membrane and the nucleoli as well as the condensation of chromosomes in the process of M phase progression of the cell cycle. In the present article, we briefly introduce the behavior of AS2 bodies during progression of the mitotic phase of cells in leaf primordia (lower panels of Fig. 8). AS2 bodies are observed around condensed chromosomes at prophase and metaphase, after which each body splits into two bodies at anaphase, and they segregate into daughter cells with the separation of chromosomes. An anaphase-bridge-like structure (bottom panel), which resembles ultrafine DNA bridges containing rDNA repeats reported in animal cells (Liu et al. 2014; Nielsen and Hickson, 2016), is visible between two separating AS2 bodies. These results imply that AS2 bodies might also play a role in the segregation of daughter chromosomes after metaphase of the cell cycle in addition to the repressive regulation of *ETT*/*ARF3* by AS2.

## A role of AS2 in virus pathogenicity in plants

It has been reported that AS1 and AS2 might play roles in the occurrence of infectious symptoms by plant viruses. βC1 protein, the pathogenicity factor of TYLCCNV (tomato yellow leaf curl china virus), interacts with AS1 protein to develop the infectious symptoms and the disease symptoms of virus-infected plants resembling the phenotypes of plants overexpressing *AS2* (Yang et al. 2008). Ye et al. (2015) also reported that the pathogenesis-related protein BV1 encoded by *Cabbage leaf curl Virus* (CaLCuV), a model for bipartite geminivirus, interacts with AS2, which is recruited to cytoplasm from the nucleus and exerts pressure on AS2 to play a novel role that favors viral infections. BV1 induces expression of the *AS2* gene by binding to its promoter even in mature plant leaves, in which *AS2* is silent, and the newly synthesized AS2 protein is first imported into the nucleus and then exported to the cytoplasmic granules, P bodies. In P bodies, AS2 interacts with one of its constituents, DCP2 (mRNA-decapping enzyme 2), to promote its decapping activity, decrease the levels of siRNAs, and attenuate PTGS (post transcriptional gene silencing), which is an endogenous critical gene repression system. In addition, overexpression of *AS2* in plants (*A. thaliana* and *N. benthamiana*) increases the susceptibility of plants to geminivirus infection. On the other hand, the *as2* mutant showed improved virus resistance. These observations are consistent with the hypothesis that PTGS acts as an important defense strategy for plants that attack the virus: the finding in the report is simply that AS2 is hijacked by BV1 in the nucleus, even in the mature leaves, is transported to the cytoplasm by the action of BV1, and contributes to the weakening of the defense network called PTGS. How AS2 could elevate the decapping activity of DCP2 by its binding in the P bodies, however, has yet to be investigated.

As we mentioned in Sect. 6 and Fig. 7c, the *as2* mutant protein (delta ICG25-80) exclusively accumulates in the cytoplasm (Luo et al. 2020), and the LZL region contains the NES-like sequence (Fig. 4). This observation allows us to predict the possibility that the LZL region might play a role in localization of AS2 to the cytoplasm under certain cellular conditions. BV1 of the geminivirus might bind to the ICG region of AS2 in the nucleus to enhance the action of a putative plant exportin to the proposed NES-like sequence in LZL, which might allow AS2 to be exported to the cytoplasm.

The studies in this section are good examples to show that plant proteins may interact with proteins derived from pathogenic microorganisms to acquire a variety of previously unknown functions. Such invisible properties of molecules might have been born through the evolution of interactions between organisms. Behind molecular interactions, many other proteins in host plants might also gain functions that have yet to be unveiled. Research on pathogenic proteins may lead to the manifestation of the hidden properties of such proteins in host cells. Interestingly, the leaf morphology of some bred vegetables belonging to the *Brassica* plants resembles the leaf phenotypes of *as2* mutants of *A. thaliana* (Col-0). In the process of vegetable breeding, genetic backgrounds of being resistant to viral infections might have spread through these plants, some of which might carry mutations in *AS2* and/or related members of the family.
